# Comprehensive Evaluation of Safety, Functional Properties, and Immunomodulatory Activity of 19 Probiotic Strains Approved by the Ministry of Food and Drug Safety (MFDS)

**DOI:** 10.4014/jmb.2605.05037

**Published:** 2026-07-07

**Authors:** Yeonju Kim, Soeun Lee, Seonju Kim, Minyeong Jung, Jieun Jung, Ju-Hoon Lee, Jaewoo Bai

**Affiliations:** 1 Department of Food Science and Biotechnology, Seoul Women’s University, Seoul 01797, Republic of Korea; 2 Department of Agricultural Biotechnology, Seoul National University, Seoul 08826, Republic of Korea

**Keywords:** Approved probiotic strain, Safety, Probiotic property, Immunomodulatory activity

## Abstract

The Ministry of Food and Drug Safety (MFDS) has approved 19 probiotic strains for use as functional food ingredients. Although the characteristics of individual strains have been previously reported, a comprehensive evaluation of all 19 MFDS-approved probiotic strains under a unified experimental framework has not yet been conducted. Therefore, this study systematically assessed the safety, probiotic properties, and immunomodulatory activity of the 19 MFDS-approved strains. Cytotoxicity, bile salt hydrolase (BSH) activity, D-lactate production, tolerance to simulated gastrointestinal tract (GIT) conditions, adhesion capacity, and immunomodulatory activity were evaluated. All strains showed no cytotoxicity toward Caco-2 cells. BSH activity was detected in 14 strains, whereas D-lactate production (1.14–28.88 mM) was observed only in *Lactobacillaceae* strains. The strains exhibited a broad range of GIT tolerance, with survival rates ranging from 23.55% to 95.58%. *Lactobacillus gasseri*, *Bifidobacterium animalis* subsp. lactis, and *Bifidobacterium breve* showed high survival rates (>80%), whereas *Bifidobacterium bifidum* and *Streptococcus thermophilus* exhibited relatively low tolerance (<40%). Adhesion capacity was generally similar among strains (59.84~87.82%), although *B. bifidum* showed the highest adhesion rate (87.82%). In contrast, *Enterococcus faecium* and *Bifidobacterium longum* subsp. *longum* exhibited relatively low adhesion capacities. Furthermore, 14 strains modulated immune responses in LPS-stimulated RAW 264.7 cells by regulating TNF-α and IL-6 secretion. Collectively, these findings demonstrate the overall safety and strain-specific functional properties of the 19 MFDS-approved probiotic strains and provide fundamental information for selecting suitable candidates for targeted probiotic applications.

## Introduction

Probiotics, defined by the Food and Agriculture Organization (FAO) and World Health Organization (WHO) in 2002 as “live microorganisms that, when administered in adequate amounts, confer a health benefit on the host”, have emerged as a promising strategy for promoting human health [1]. Probiotics exert their beneficial effects through tri-directional interactions among the host and gut microbiota, including modulation of the gut microbial composition, immunomodulation, reinforcement of the intestinal epithelial barrier, and biosynthesis of organic acids [2]. Lactic acid bacteria (LAB) are widespread and represent one of the most important bacterial groups in the food industry. Many LAB are recognized as microorganisms with Qualified Presumption of Safety (QPS) and/or Generally Recognized as Safe (GRAS) status. Currently, LAB strains used as probiotics include members of the genera *Lactobacillus*, *Lactococcus*, *Leuconostoc*, *Bifidobacterium*, *Streptococcus*, *Pediococcus*, *Weissella*, and *Enterococcus* [3-5].

The selection of probiotic microorganisms requires a systematic and stepwise approach due to the large number of isolated strains that must be progressively screened to identify potential probiotic candidates [6]. Probiotic LAB must demonstrate resilience to gastrointestinal stresses, including low pH, digestive enzymes, bile salts, phenolic compounds, and heat shock. They should also exhibit the ability to adhere to intestinal epithelial cells—often reflected by auto-aggregation and hydrophobicity—and to inhibit pathogens through the production of antimicrobial metabolites. Safety assessment is likewise critical and includes confirmation of taxonomic identity, the absence of virulence traits, minimal antibiotic resistance, and suitability for incorporation into daily diets [3].

Probiotics, traditionally associated with health benefits, are now widely recognized for their applications in various industrial processes, including the dairy industry, functional foods, bioactive compounds, cosmetics, pharmaceuticals, bioremediation and waste management, agriculture, and animal feed supplements [5]. The consumption of probiotics has increased substantially, and the Korean probiotic market has grown continuously, expanding from 217.4 billion won in 2017 to 525.6 billion won in 2020 based on annual sales data [7]. In Korea, the Ministry of Food and Drug Safety (MFDS) has approved 9 genera and 19 species of microorganisms as functional ingredients for use in health-promoting foods. These include *Lactobacillus* (*L. acidophilus*, *L. gasseri*, *L. delbrueckii* subsp. *bulgaricus*, *L. helveticus*), *Lacticaseibacillus* (*Lcb. casei*, *Lcb. paracasei*, *Lcb. rhamnosus*), *Limosilactobacillus* (*Lmb. fermentum*, *Lmb. reuteri*), *Lactiplantibacillus* (*Lp. plantarum*), *Ligilactobacillus* (*Lg. salivarius*), *Lactococcus* (*Lc. lactis*), *Enterococcus* (*E. faecium*, *E. faecalis*), *Streptococcus* (*S. thermophilus*), and *Bifidobacterium* (*B. bifidum*, *B. breve*, *B. longum*, *B. animalis* subsp. *lactis*) [7, 8]. For approval as functional ingredients, probiotic strains must undergo comprehensive safety evaluations. The MFDS outlines specific assessment procedures, including tests for antibiotic resistance, hemolytic activity, cytotoxicity, and metabolic characteristics such as D-lactate production and bile salt deconjugation [8]. Previous studies have reported that isolated strains, including *Lg. salivarius* AP-32, *Lp. plantarum* ZFM55, *Lg. salivarius* OSTIA SALI-10, *Lmb. reuteri* ID-D01, and *E. faecium* KU22001, were evaluated for probiotic potential and safety through analyses of hemolytic and enzymatic activities, bile salt deconjugation, acid and bile salt tolerance, D-/L-lactate production, cytotoxicity, antibiotic susceptibility, adhesion ability, and cell surface hydrophobicity, among others parameters [1, 2, 9-11].

However, most previous studies have focused on isolated or individual strains; to date, no comprehensive study has systematically evaluated all 19 probiotic strains officially recognized by the MFDS. Therefore, the present study comprehensively evaluated the safety profiles, probiotic properties, and immunomodulatory properties of all 19 probiotic strains approved by the MFDS. This study provides a framework for the comprehensive evaluation of newly isolated probiotic strains.

## Materials and Methods

### Bacterial Strains and Culture Conditions

The probiotic strains used in this study were obtained from the American Type Culture Collection (ATCC, USA). A total of 19 strains were selected based on the probiotic species listed in the notification of the Korean Ministry of Food and Drug Safety (MFDS). For each species, the type strain registered in ATCC was used as a representative strain and detailed information on the strains are provided in Table 1. All strains were stored at -80°C in 15% (v/v) glycerol stocks and cultured twice for 18 h under their respective optimal growth conditions prior to use.

### Cytotoxicity to Caco-2 Cells

Caco-2 human colorectal adenocarcinoma cells were cultured in Dulbecco’s Modified Eagle’s Medium (DMEM; Welgene, Republic of Korea) containing 10% fetal bovine serum (FBS; Gibco, Canada) at 37°C in a humidified atmosphere containing 5% CO_2_. The cytotoxicity of the probiotic strains was assessed using the CytoTox 96^®^ Non-Radioactive Cytotoxicity Assay kit (Promega, USA) according to the manufacturer’s instructions and specific assay conditions were established based on our preliminary studies. Briefly, Caco-2 cells were seeded into 96-well plates at a density of 5 × 10^5^ cells/mL and incubated at 37°C for 24 h. After incubation, probiotic strains were added to each well at a density of 1 × 10^9^ CFU/mL and incubated for an additional 24 h. DMEM-treated wells were used as the negative control, while wells treated with the lysis solution served as a positive control. Following incubation, the plates were centrifuged at 600 × g for 6 min, and the supernatants were collected. Each supernatant was mixed with the LDH substrate solution and incubated in the dark at room temperature for 20 min. Absorbance was measured at 490 nm using a microplate reader (SpectraMax M2 Microplate Reader, Molecular Devices, USA), and cytotoxicity (%) was calculated as follows [12]:

$$\text{Cytotoxicity}\left(\%\right)=\frac{{\text{Absorbance}}_{\text{sample}}-{\text{Absorbance}}_{\text{negative control}}}{{\text{Absorbance}}_{\text{positive control}}-{\text{Absorbance}}_{\text{negative control}}}\times 100$$

### Bile Salt Deconjugation

Bile salt hydrolase (BSH) activity of probiotic strains was assessed using a plate-based taurodeoxycholic acid (TDCA; Sigma-Aldrich, USA) assay, following previously described methods [13-15]. Different concentrations of TDCA and cysteine were applied based on the physiological characteristics of each species, as determined by our preliminary tests. Specifically, *L. bulgaricus* and *S. thermophilus* were evaluated at a lower TDCA concentration (0.15%, w/v), as these species are known to have limited bile tolerance, and higher concentrations resulted in complete growth inhibition, preventing accurate evaluation of BSH activity [16, 17]. All other strains were tested at 0.5% TDCA (w/v). In addition, cysteine was supplemented at a final concentration of 0.05% (w/v) exclusively for *Bifidobacterium* strains, as these are obligate anaerobes that require reducing conditions for optimal growth [18]. Each strain was streaked onto TDCA-containing agar plates and incubated under anaerobic conditions at 37°C for 72 h. Positive BSH activity was determined by the formation of precipitation halos surrounding bacterial colonies.

### D-Lactate Assay

D-lactate levels were measured using a D-lactate colorimetric assay kit (Abcam, UK). Briefly, all probiotic cultures were centrifuged at 11,000 × g for 10 min at 4°C. Then, the supernatants were treated with 4 M perchloric acid (PCA; Sigma-Aldrich) for deproteinization. Then, the supernatants subsequently neutralized to pH 6.5-8.0 using 0.1 and 2 M potassium hydroxide (KOH; Samchun Chemical, Republic of Korea). Finally, each sample was subjected to the assay according to the manufacturer’s instructions and absorbance was measured at 450 nm using a microplate reader (SpectraMax M2 Microplate Reader, Molecular Devices) [19].

### Tolerance to Simulated Gastrointestinal Tract

Simulated gastric and intestinal fluids were prepared according to previously described methods [20] to evaluate the survival of probiotic strains under simulated gastrointestinal tract (GIT) conditions. Simulated gastric fluid (SGF) was prepared by dissolving NaCl (125 mM), KCl (7 mM), and NaHCO_3_ (45 mM) in sterilized distilled water. Pepsin (Sigma-Aldrich) was added at a concentration of 3 g/L, and the pH was adjusted to 2.5 using 5 N HCl. The SGF was inoculated with probiotic strains at a final concentration of approximately 1 × 10^8^ CFU/mL and incubated at 37°C for 2 h. Following the gastric phase, simulated intestinal fluid (SIF) was added to the SGF at a 1:1 (v/v) ratio. Pancreatin and bovine bile salts (Sigma-Aldrich) were added at final concentrations of 0.1% and 0.15% (w/v), respectively. The pH was then adjusted to 8.0 using 5 N NaOH and incubated at 37°C for 3 h to simulate the intestinal phase. During GIT incubation, *Bifidobacterium* strains were incubated under anaerobic conditions as they are obligate anaerobes, whereas all other strains were incubated aerobically. After SGF–SIF incubation, aliquots were collected, and each strain was plated on its respective optimal culture medium (Table 1). Viable bacterial cells were counted to determine survival rates. The survival rate (%) was calculated according to the following Equation [21]:

$$\text{Survival rate}\left(\%\right)=\frac{\text{Bacterial number after incubation}\left(\text{Log CFU/mL}\right)}{\text{Initial bacterial number}\left(\text{Log CFU/mL}\right)}\times 100$$

### Adhesion to Caco-2 Cells

Caco-2 cells were cultured in Minimum Essential Medium Eagle (MEM; Welgene) supplemented with 10% FBS at 37°C in a humidified atmosphere containing 5% CO_2_. Cells were seeded into 24-well plates at a density of 5 × 10^5^ cells/mL and allowed to grow for 14 days to obtain a monolayer of differentiated cells [22]. During the differentiation period, the culture medium was replaced every 2­3 days with serum-free MEM. Prior to bacterial inoculation, the culture medium was removed, and the cell monolayers were washed with phosphate-buffered saline (PBS; BTBKorea Co., Ltd., Republic of Korea). For the adhesion assay, probiotic strains were added to the wells at a concentration of approximately 1 × 10^8^ CFU/mL and incubated for 90 min [23]. After incubation, the cells were washed three times with PBS to remove non-adherent bacteria. The cells were then detached by treatment with 1 mL of 0.25% trypsin–EDTA solution (Thermo Fisher Scientific, USA) for 10 min. The resulting suspension was collected, serially diluted, and the number of adhered bacteria was determined by plating on the respective optimal culture media for each strain, as detailed in Table 1. The adhesion rate (%) was calculated as follows [24]:

$$\text{Adhesion rate}\left(\%\right)=\frac{\text{Attached bacterial number}\left(\text{Log CFU/mL}\right)}{\text{Initial bacterial number}\left(\text{Log CFU/mL}\right)}\times 100$$

### Immunomodulatory Effects in Lipopolysaccharide (LPS)-Stimulated RAW 264.7

RAW 264.7 murine macrophages were cultured in DMEM supplemented with 10% FBS at 37°C in a humidified atmosphere containing 5% CO_2_. Cells were seeded into a 96-well plate at a density of 5 × 10^5^ cells/mL and incubated at 37°C for 24 h. The cells were pretreated with each probiotic strain at concentrations ranging from 10^6^ to 10^8^ CFU/mL for 2 h, followed by stimulation with LPS from *Escherichia coli* O111:B4 (Invitrogen, USA) at a final concentration of 0.1 μg/mL. For controls, cells treated with DMEM alone were used as a negative control (NC), while cells stimulated with LPS alone served as the positive control (PC). In addition, dexamethasone (DEX), known to suppress pro-inflammatory cytokine production, was included as a reference control [25]. After incubation for an additional 22 h, culture supernatants were collected by centrifugation (600 × g, 6 min). Cytotoxicity of the bacterial treatments was evaluated using CytoTox 96^®^ Non-Radioactive Cytotoxicity Assay kit, and non-cytotoxic concentrations of the bacterial strains were determined to exclude potential cytotoxic effects on immunomodulatory properties. The levels of proinflammatory cytokines, tumor necrosis factor-α (TNF-α) and interleukin-6 (IL-6), were then quantified in the culture supernatants using DuoSet ELISA kits (R&D Systems, USA) according to the manufacturer’s instructions. Cytokine levels were expressed as percentages of the positive control and calculated using the following equation [26]:

$$\text{Cytokine secretion}\left(\%\right)=\frac{\text{Cytokine level in sample}}{\text{Cytokine level in positive control}}\times 100$$

### Statistical Analysis

All experiments were performed in triplicate (n = 3), and data are presented as mean ± standard deviation (SD). Statistical significance was determined using one-way analysis of variance (ANOVA) followed by Dunnett’s test, performed using IBM SPSS Statistics version 29.0.2.0 (IBM Corp., USA).

## Results and Discussion

### Cytotoxicity of 19 MFDS Strains in Caco-2 Cells

**Fig. 1:**
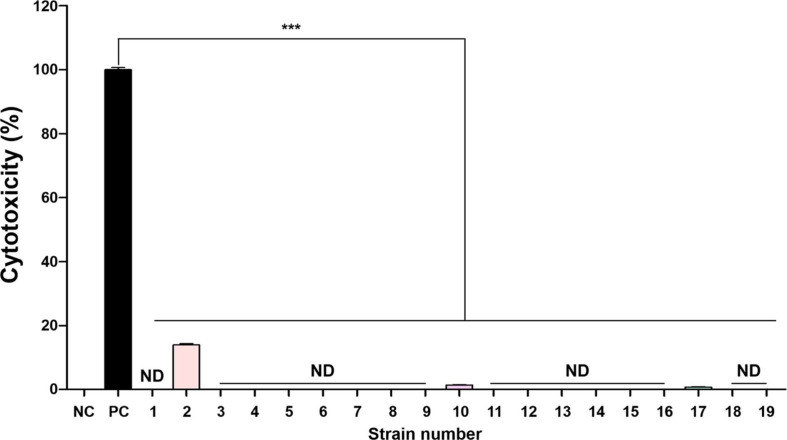
Cytotoxicity of 19 MFDS strains in Caco-2 cells. NC, negative control; PC, positive control. ND, not detected. Data are expressed as mean ± SD. Statistical significance was determined by one-way ANOVA followed by Dunnett's multiple comparison test with the positive control (***P < 0.001).

To evaluate the safety of 19 MFDS strains, an *in vitro* cytotoxicity assay was conducted using Caco-2 cells. As shown in Fig. 1, none of the 19 tested probiotic strains exhibited significant cytotoxicity. The cytotoxicity levels of *L. bulgaricus*, *Lmb. fermentum*, and *E. faecium* were 13.93, 1.65, and 0.95%, respectively. Importantly, all measured cytotoxicity values were below 20%, indicating that the tested strains were non-cytotoxic under the experimental conditions. These findings are consistent with previous reports demonstrating that *Lmb. reuteri* ID-D01, *Lg. salivarius* AP-32 and *Lp. plantarum* UTNGt3 did not induce cytotoxicity to Caco-2 cells [2, 9, 12]. Collectively, the results support the *in vitro* safety profile of 19 MFDS strains with respect to epithelial cell viability.

### Bile Salt Hydrolase Activity of 19 MFDS Strains

BSH activity plays a pivotal role in cholesterol metabolism, and its expression in probiotic strains confers resistance to bile salts, thereby facilitating intestinal colonization [8, 27]. The BSH activity of 19 MFDS strains was evaluated using a plate-based TDCA assay and activity was determined by the formation of precipitated halos or opaque colonies surrounding the bacterial growth. As shown in Table 2, 14 strains exhibited detectable BSH activity, whereas 5 strains showed no measurable BSH activity under the tested conditions. Consistent with previous findings, *Lc. lactis* ATCC 8000 showed no bile salt deconjugation in TDCA, while *L. acidophilus* ATCC 832, *Lp. plantarum* ATCC 4008, and *Lg. salivarius* AP25a exhibited BSH activity under similar conditions [15]. Overall, these results indicate that BSH activity among 19 MFDS strains is widely distributed, suggesting their potential role in bile acid metabolism and intestinal adaptation [8, 27].

### D-Lactate Production by 19 MFDS Strains

**Fig. 2:**
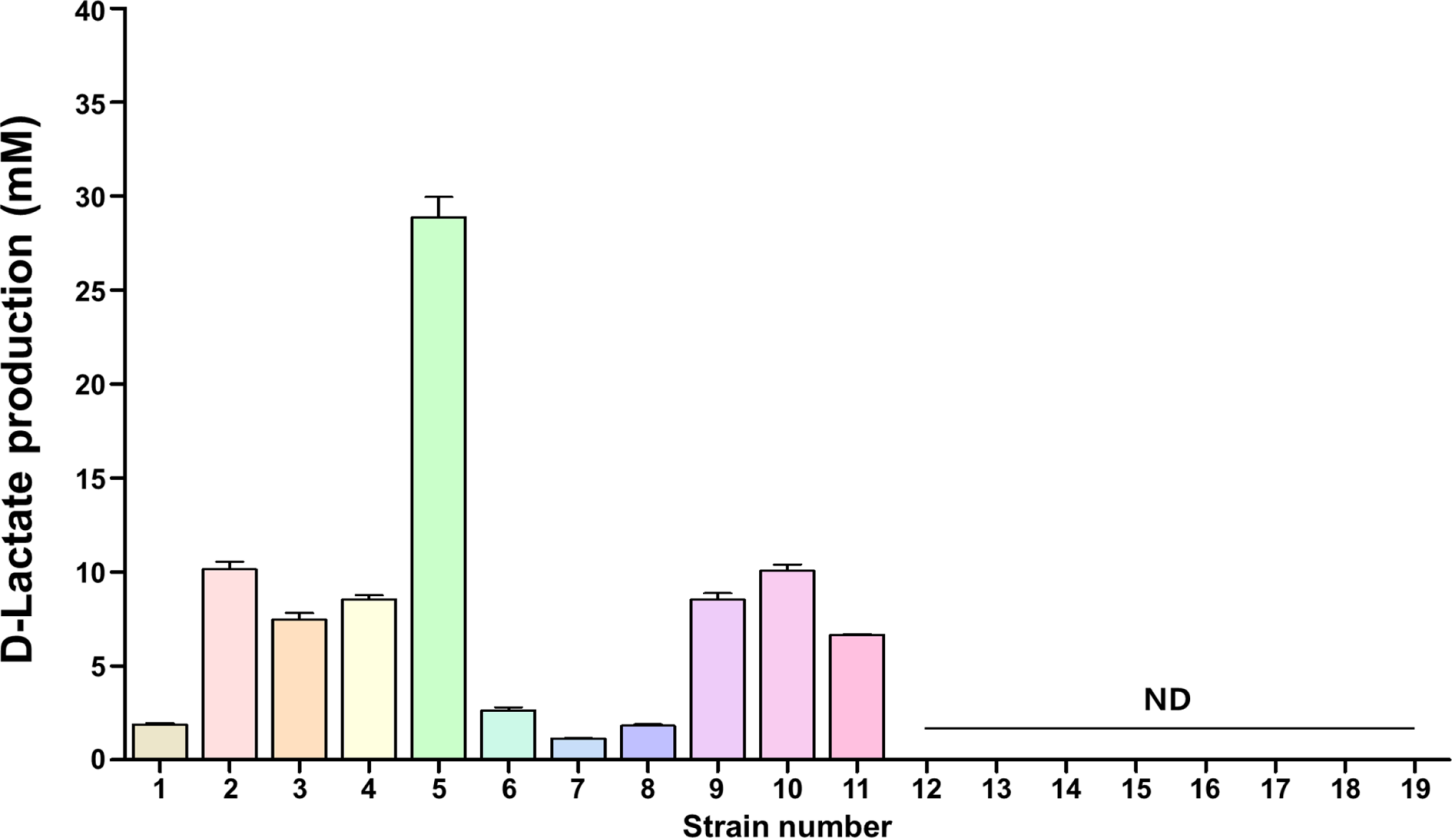
D-Lactate concentration produced by 19 MFDS strains. ND, not detected. Data are presented as mean ± SD.

Lactic acid bacteria can produce L-, D-, DL-lactate, or combination of these depending on their metabolic pathways [28]. Notably, D-lactate is

metabolized more slowly than L-lactate in humans, and its accumulation has been implicated as a potential risk factor for D-lactic acidosis, particularly in patients with short bowel syndrome. Accordingly, the WHO has emphasized the importance of limiting its intake [2]. Therefore, the D-lactate production levels of 19 MFDS strains were evaluated. As shown in Fig. 2, D-lactate production in 11 strains of the *Lactobacillaceae* family (No.1-11) ranged from 1.14 to 28.88 mM. In contrast, 8 strains (No. 12-19), including members of *Bifidobacterium* spp., *Enterococcus* spp., *Lc. lactis*, and *S. thermophilus* did not produce detectable levels of D-lactate. While genera such as *Bifidobacterium* spp., *Enterococcus*, *Lc. lactis*, and *S. thermophilus* primarily produce L-lactate, members of the *Lactobacillaceae* family are capable of producing L-, D-, and DL-lactate. Consequently, they are characterized by relatively high D-lactate production, and the 19 strains were consistent with previous studies [29]. Furthermore, members of the *Lactobacillaceae* family such as *L. acidophilus*, *Lmb. reuteri*, and *Lmb. fermentum* have been reported to produce diverse levels of D-lactate depending on the specific strain, origin, and culture conditions [29-31]. Given the differences in D-lactate production for each strain, a comprehensive safety assessment of probiotics requires evaluation at the strain level, beyond species-level.

### Survival of 19 MFDS Strains under Simulated Gastrointestinal Conditions

**Fig. 3:**
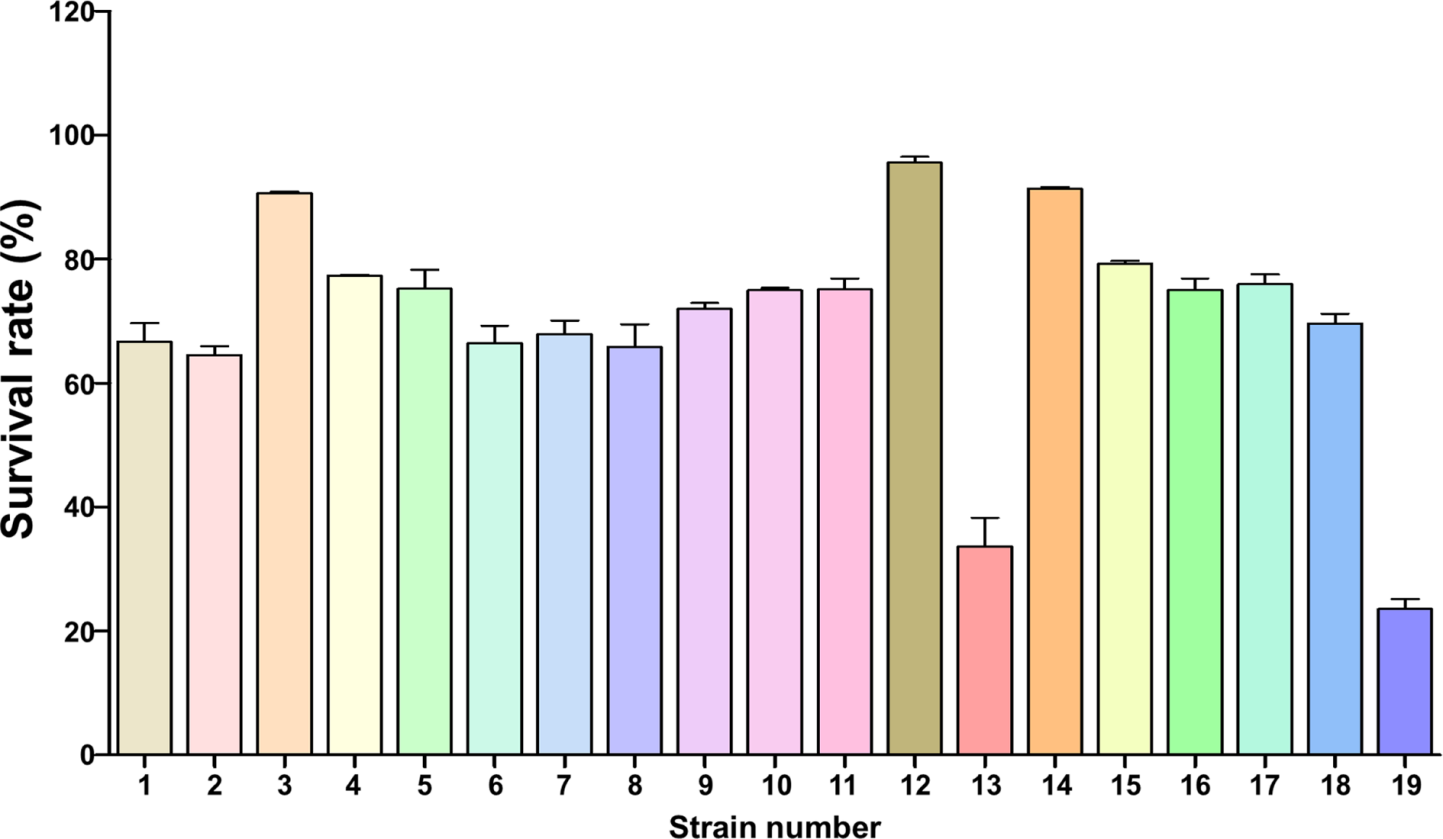
Tolerance of 19 MFDS strains to simulated gastrointestinal conditions. Data are presented as mean ± SD.

Survival under acidic pH, bile salts, gastrointestinal conditions is widely recognized as a critical criterion for probiotic selection, as bacteria must withstand harsh physiological environments to reach their target site in the GIT [20]. Accordingly, the 19 MFDS strains were subjected to simulated GIT conditions, and the survival rates were evaluated (Fig. 3). Under sequential gastrointestinal exposure conditions, 17 of 19 strains maintained relatively high viability, with survival rates ranging from 64.53 to 95.58%. Consistent with these findings, previous studies have reported that strains such as *B. animalis* ssp. *lactis* (ATCC 27536, HY8002) and *B. breve* (HY3016, HY8921) also exhibit similarly high viability (>80%) under simulated GIT conditions [32, 33]. In contrast, *B. bifidum* and *S. thermophilus* showed significantly lower survival rates of 33.62% and 23.55%, respectively. This is consistent with previous reports showing that *B. bifidum* exhibited relatively poor tolerance under simulated GIT conditions, with survival rates ranging from approximately 0% to 20% [34]. Similarly, *S. thermophilus* has been reported to exhibit limited tolerance to gastrointestinal stress, with survival rates typically below 10% following sequential exposure to GIT conditions [35]. While most of the 19 MFDS strains generally possess substantial tolerance to gastrointestinal stress, the observed variability underscores the importance of species-level characterization when selecting potent probiotic candidates.

### Adhesion Capacity of 19 MFDS Strains to Caco-2 Cells

**Fig. 4:**
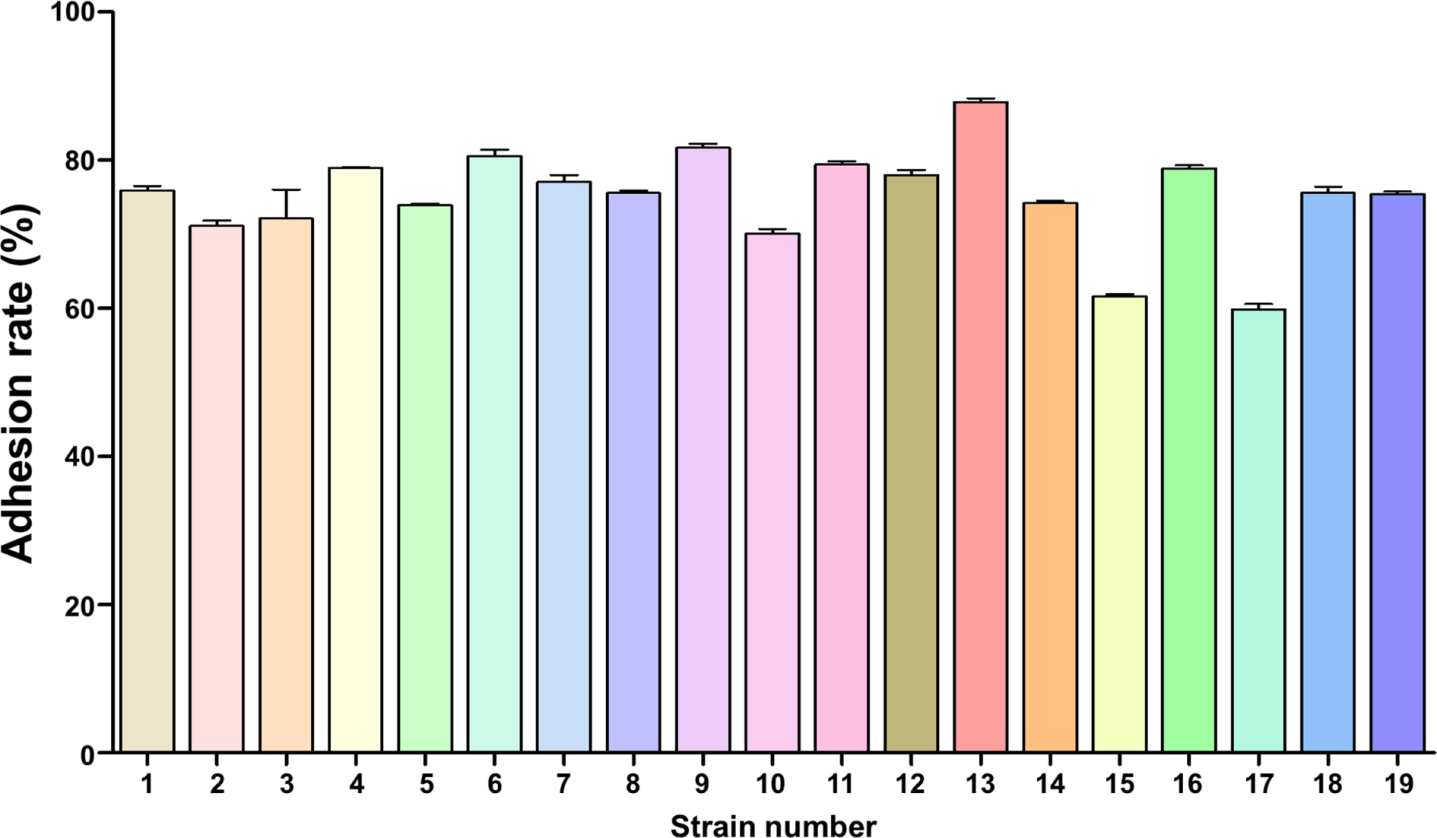
*In vitro* adherence of 19 MFDS strains to Caco-2 cells. Data are presented as mean ± SD.

Adhesion to intestinal epithelial cells is a critical determinant of probiotic colonization and persistence in the gastrointestinal tract, contributing to their functional efficacy and making it an important parameter for strain evaluation [1, 36]. Previous studies have reported that the adhesion ability of probiotic strains to intestinal epithelial cells varies in a species-specific manner. This variability is attributed to differences in cell surface components, such as exopolysaccharides (EPS), surface proteins, and lipoteichoic acids, which interact with epithelial cell receptors. Other factors, including auto-aggregation, surface hydrophobicity, and co-aggregation, have also been shown to influence adhesion, reflecting species-specific surface structures and mechanisms [37]. The adhesion capacities of the 19 MFDS strains to Caco-2 cells are presented in Fig. 4. Adhesion rates ranged from 59.84 to 87.82%, with *B. bifidum* exhibiting the highest adhesion rate (87.82%) and *E. faecium* showing the lowest (59.84%). Notably, *B. bifidum* strains have been reported to exhibit strong adhesion to intestinal epithelial cells, which has been associated with the presence of surface-associated proteins such as the lipoprotein BopA that mediates host cell interaction [38, 39]. These findings support the high adhesion capacity observed for *B. bifidum* in the present study. Furthermore, comparative studies among bifidobacterial strains have shown that *B. longum* E18 and *B. breve* BBSF exhibit relatively lower adhesion abilities than *B. bifidum* strains (*e.g.*, S16 and S17) and *B. lactis* strains (*e.g.*, L15 and BI07), supporting the species-dependent differences in adhesion observed in this study [40]. In contrast, *E. faecium*, which showed the lowest adhesion rate (59.84%), has been reported to exhibit similar levels of adhesion (approximately 60%) in strains isolated from fermented sausages, indicating that despite relatively lower adhesion compared to other strains, it still possesses acceptable probiotic adhesion capacity [41]. Overall, these results indicate that 19 MFDS strains exhibit varying but generally substantial adhesion capacities, highlighting their potential for intestinal interaction and persistence.

### Immunomodulatory Effects of 19 MFDS Strains on Pro-Inflammatory Cytokine Production in LPS-Stimulated RAW 264.7 Cells

**Fig. 5:**
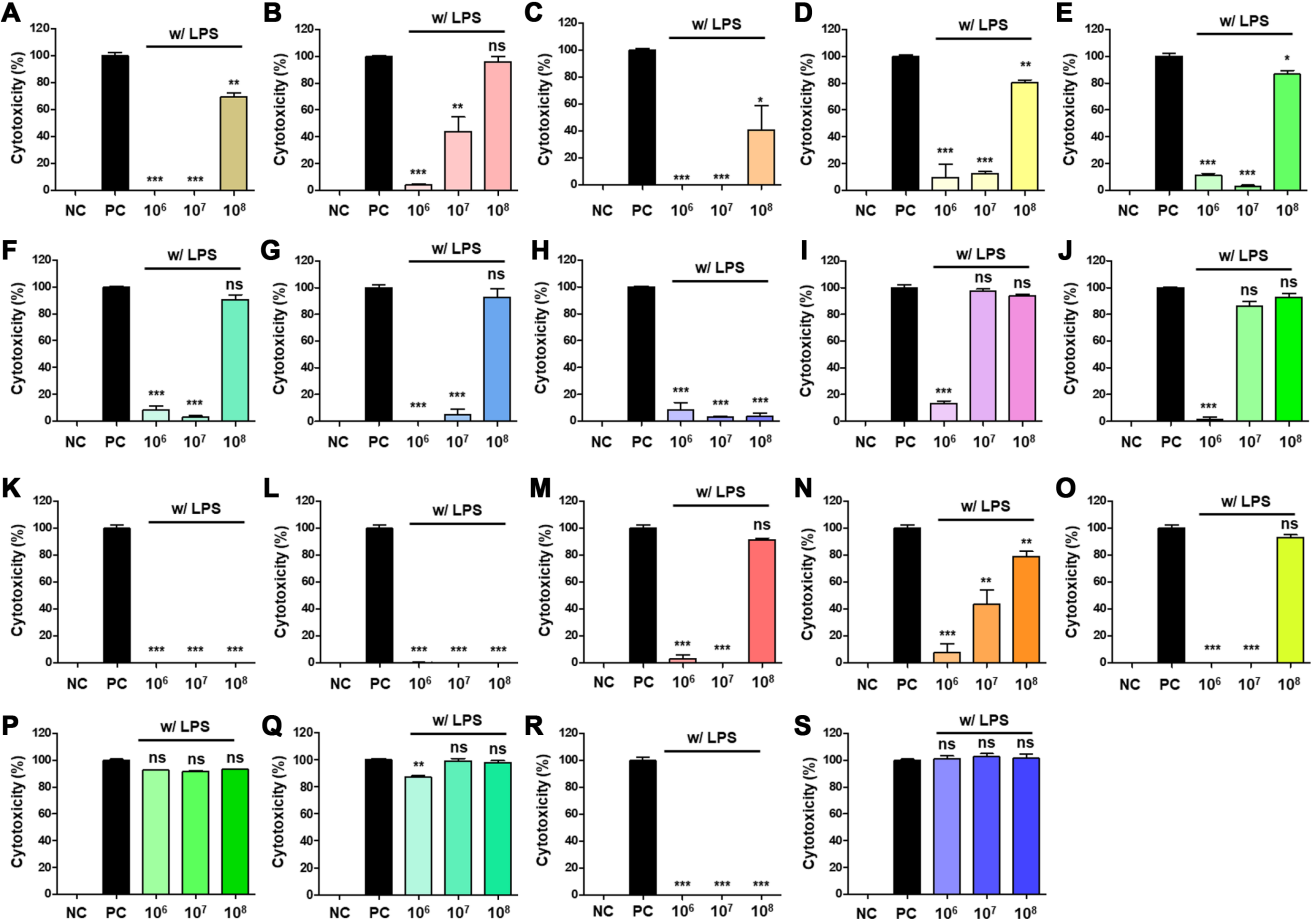
Cytotoxicity of 19 MFDS strains in LPS-stimulated RAW 264.7 cells. Panels (A–S) correspond to strains number 1–19, respectively. NC, negative control; PC, positive control. Data are expressed as mean ± SD. Statistical significance was determined by one-way ANOVA followed by Dunnett's multiple comparison test compared with the positive control. compared with the positive control. ns, not significant; * *P* < 0.05; ** *P* < 0.01; *** *P* < 0.001.

**Fig. 6:**
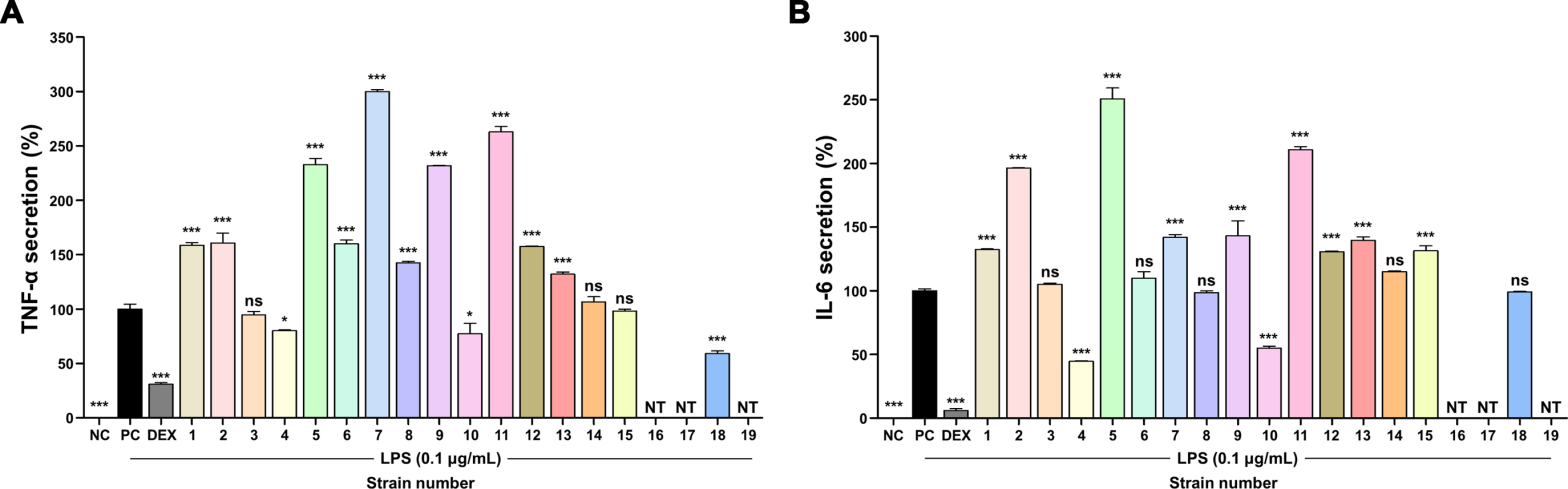
Immunomodulatory effects of 19 MFDS strains on TNF-α (A) and IL-6 (B) secretion in LPS-stimulated RAW 264.7 cells. NT, not tested. NC, negative control; PC, positive control; DEX, dexamethasone. Data are presented as mean ± SD. Statistical significance was determined by one-way ANOVA followed by Dunnett’s multiple comparison test. Asterisks (* *P* < 0.05, ** *P* < 0.01, *** *P* < 0.001) indicate significant differences compared with the positive control, while ns indicates no significant difference.

Macrophages play a central role in innate immunity by secreting pro-inflammatory mediators including TNF-α and IL-6, which contribute to the regulation of host defense responses [42]. While moderate levels of these cytokines contribute to host protection, their excessive or dysregulated production may lead to pathological inflammation [43]. Probiotic bacteria have been reported to modulate host immunity by enhancing macrophage activation and altering cytokine profiles, with these effects being highly strain dependent [44, 45]. Given these strain-specific characteristics, accurate evaluation of immunomodulatory effects is essential. However, some previous studies have reported that certain bacteria reduce pro-inflammatory responses and exhibit anti-inflammatory effects without evaluating cytotoxicity under the same experimental conditions used for immunomodulatory evaluation, suggesting that the observed reduction in inflammation may have been influenced by unaccounted cytotoxic effects [46, 47]. Thus, to prevent misinterpretation of immunomodulatory activity, an LDH assay was first performed to determine non-cytotoxic bacterial concentrations, followed by immune assays using only those concentrations for all 19 strains (Figs. 5 and 6) [48]. As shown in Fig. 5, three of the 19 strains (*S. thermophilus*, *E. faecium*, and *E. faecalis*) exhibited cytotoxicity at all tested concentrations and were therefore excluded from subsequent immune assays. For the remaining strains, non-cytotoxic concentrations (10^5^–10^7^ CFU/mL) were selected and used for further immune assays. Using these non-cytotoxic concentrations, the immunomodulatory effects of the probiotic strains were evaluated by measuring TNF-α and IL-6 secretion in LPS-stimulated RAW 264.7 cells (Fig. 6A and 6B). As a reference control, dexamethasone (DEX) significantly reduced cytokine production, with TNF-α and IL-6 levels decreasing to 31.01% and 6.12% of the positive control, respectively. In cells treated with probiotic strains, TNF-α secretion ranged from 59.23 to 299.94%, whereas IL-6 levels ranged from 44.64 to 250.81%, relative to the positive control. Among these strains, *Lcb. rhamnosus* markedly enhanced cytokine production, increasing TNF-α and IL-6 levels to 299.94% and 141.95%, respectively. These results are consistent with previous studies indicating that *Lcb. rhamnosus* can stimulate macrophage activation and induce the production of pro-inflammatory cytokines [49, 50]. Similarly, *L. bulgaricus* and *Lmb. reuteri* increased TNF-α production to 160.90% and 231.79%, respectively, which is also in accordance with previous findings [42, 50]. In contrast, several strains exhibited a modulatory effect characterized by a reduction in cytokine levels. *L. helveticus* and *Lmb. fermentum* decreased both TNF-α (80.20% and 77.31%, respectively) and IL-6 (44.64% and 55.01%, respectively) compared to the positive control, suggesting their potential to attenuate excessive inflammatory responses. Additionally, *Lc. lactis* reduced TNF-α production to 59.23%, while IL-6 levels remained comparable to the positive control (99.15%), indicating selective modulation of cytokine production rather than a broad suppressive effect. However, other studies have reported differing results at the species level. For example, *Lp. plantarum* Z22 and *Lmb. reuteri* LM1071 have been shown to reduce TNF-α and IL-6 expression, and *B. bifidum* BGN4 can suppress TNF- α and IL-6 secretion [51-53]. These contrasting results highlight that immunomodulatory effects can vary significantly even within the same species, supporting the previous findings that such effects are highly strain-specific [54, 55]. Collectively, these findings demonstrate that the tested strains exhibit distinct, strain-specific immunomodulatory profiles. These effects appear to contribute to immune homeostasis through the regulation of cytokine responses, rather than inducing uniform pro- or anti-inflammatory activities. Furthermore, certain strains may influence innate immune responses via macrophage activation and cytokine modulation, although further in vivo studies are required to validate these effects.

## Conclusion

The Ministry of Food and Drug Safety (MFDS) has approved 19 probiotic strains for use as functional food ingredients. In the present study, these strains were systematically evaluated for their safety, probiotic properties, and immunomodulatory activity under a unified experimental framework, with assay conditions optimized for each strain based on preliminary studies. All tested strains were non-cytotoxic to Caco-2 cells, confirming their overall safety, whereas species-dependent variability was observed in BSH activity and D-lactate production. In addition, the strains exhibited a wide range of tolerance to simulated gastrointestinal conditions and varying adhesion capacities to intestinal epithelial cells, suggesting differences in their potential for gastrointestinal survival and colonization. Furthermore, most of the tested strains demonstrated distinct immunomodulatory effects by differentially increasing or decreasing TNF-α and IL-6 production in LPS-stimulated RAW 264.7 cells. Collectively, these findings demonstrate the overall safety and species-specific functional and immunomodulatory properties of 19 MFDS strains, providing a basis for selecting suitable probiotic candidates for targeted applications based on strain-level characteristics.

## Figures and Tables

**Table 1:** 19 MFDS strains used in this study.

Family	Strain number	Strain	Culture condition
Lactobacillaceae	1	*Lactobacillus acidophilus* ATCC 4356	37°C, MRS, Aerobic
	2	*Lactobacillus delbrueckii* subsp. *bulgaricus* ATCC 11842	
	3	*Lactobacillus gasseri* ATCC 33323	
	4	*Lactobacillus helveticus* ATCC 15009	
	5	*Lactiplantibacillus plantarum* ATCC 8014	
	6	*Lacticaseibacillus casei* ATCC 393	
	7	*Lacticaseibacillus rhamnosus* ATCC 53103	
	8	*Lacticaseibacillus paracasei* subsp. *paracasei* ATCC 27216	
	9	*Limosilactobacillus reuteri* ATCC 23272	
	10	*Limosilactobacillus fermentum* ATCC 14931	
	11	*Ligilactobacillus salivarius* ATCC 11741	
Bifidobacteriaceae	12	*Bifidobacterium animalis* subsp. *lactis* ATCC 27673	37°C, MRS + 0.05% (w/v) L-cysteine, Anaerobic
	13	*Bifidobacterium bifidum* ATCC 29521	
	14	*Bifidobacterium breve* ATCC 15700	
	15	*Bifidobacterium longum* subsp. *longum* ATCC 15707	
Enterococcaceae	16	*Enterococcus faecalis* ATCC 19433	37°C, MRS, Aerobic
	17	*Enterococcus faecium* ATCC 19434	
Streptococcaceae	18	*Lactococcus lactis* subsp. *lactis* ATCC 19435	30°C, M17, Aerobic
	19	*Streptococcus thermophilus* ATCC 19258	37°C, M17, Aerobic

**Table 2:** Bile salt deconjugation activity of 19 MFDS strains.

Strain number	Strain	BSH Activity^a^
1	*Lactobacillus acidophilus* ATCC 4356	+
2	*Lactobacillus delbrueckii* subsp. *bulgaricus* ATCC 11842	–
3	*Lactobacillus gasseri* ATCC 33323	+
4	*Lactobacillus helveticus* ATCC 15009	+
5	*Lactiplantibacillus plantarum* ATCC 8014	+
6	*Lacticaseibacillus casei* ATCC 393	–
7	*Lacticaseibacillus rhamnosus* ATCC 53103	+
8	*Lacticaseibacillus paracasei* subsp. *paracasei* ATCC 27216	–
9	*Limosilactobacillus reuteri* ATCC 23272	+
10	*Limosilactobacillus fermentum* ATCC 14931	+
11	*Ligilactobacillus salivarius* ATCC 11741	+
12	*Bifidobacterium animalis* subsp. *lactis* ATCC 27673	+
13	*Bifidobacterium bifidum* ATCC 29521	+
14	*Bifidobacterium breve* ATCC 15700	+
15	*Bifidobacterium longum* subsp. *longum* ATCC 15707	+
16	*Enterococcus faecalis* ATCC 19433	+
17	*Enterococcus faecium* ATCC 19434	+
18	*Lactococcus lactis* subsp. *lactis* ATCC 19435	–
19	*Streptococcus thermophilus* ATCC 19258	–

^a^+, BSH activity is positive; –, BSH activity is negative.
